# When periphery rules: Enhanced sampling weights of the visual periphery in crowding across dimensions

**DOI:** 10.3758/s13423-024-02580-7

**Published:** 2024-09-20

**Authors:** Amit Yashar, Marisa Carrasco

**Affiliations:** 1 Department of Special Education, Faculty of Education, University of Haifa, 199 Abba Khoushy Ave, 3498838 Haifa, Israel; 2 Department of Psychology and Center for Neural Science, New York University, New York, NY USA

**Keywords:** Visual periphery, Crowding, Inner-outer asymmetry, Object recognition

## Abstract

Crowding, our inability to identify a feature or object – the target – due to its proximity to adjacent features or objects – flankers – exhibits a notable inner-outer asymmetry. This asymmetry is characterized by the outer flanker – more peripheral – creating stronger interference than the inner one – closer to the fovea. But crowding is not uniform across different feature dimensions. For example, in the case of orientation, this asymmetry reflects misreport errors: observers are more likely to misidentify the outer flanker as the target than the inner one. However, for spatial frequency (SF), observers tend to average the features of the target and flankers (Yashar et al., 2019). Here, we investigated whether and how the inner-outer asymmetry manifests across various feature dimensions: Gabor orientation and SF, as well as T-shape tilt and color. We reanalyzed continuous estimation reports data published by Yashar et al. (2019), focusing on a previously unanalyzed factor: the relative position of each flanker (inner vs. outer). We fit probabilistic models that assign variable weights to each flanker. Our analysis revealed that observers predominantly misreport the outer flanker as the target with Gabor orientation and T-shape tilt stimuli, and slightly so with color stimuli, whereas with Gabor SF, observers perform a weighted average of all features but also with a bias towards the outer flanker over the inner one. These findings suggest that an increased weighting on the more peripheral items is a general characteristic of crowding in peripheral vision.

## Introduction

When fixating our gaze on a single point, we seem to perceive the visual environment as a continuous, high-resolution, and coherent image. However, attempting to describe objects outside the center of vision dispels this illusion, revealing the limitations of our visual representation in the periphery (Whitney & Levi, 2011). A fundamental constraint on peripheral vision is the phenomenon known as *crowding*, which hinders our ability to identify a target object due to its proximity to nearby objects (flankers) (Levi, 2008; Pelli et al., 2004; Pelli & Tillman, 2008; Whitney & Levi, 2011). Specifically, when objects are too closely spaced, below the critical crowding spacing (Bouma, 1970), they become indistinguishable, fundamentally limiting object recognition. Crowding has implications beyond simple perception, affecting essential functions, such as reading, and contributing to visual disorders, like amblyopia (Hussain et al., 2012) and macular degeneration (Wallace et al., 2017). Thus, understanding crowding has both theoretical and translational applications.

Visual crowding is not a unitary phenomenon; it occurs across different levels of object representation, from basic features such as orientation and color to more complex objects like letters, faces, and words. It can be moderated by global-level configurations and representations, including Gestalt principles (Jimenez et al., 2022), grouping (Herzog et al., 2015; Livne & Sagi, 2010), and objecthood (Kimchi & Pirkner, 2015; Manassi & Whitney, 2018), as well as even higher-order factors such as perceptual learning (Hussain et al., 2012; Plank et al., 2021; Yashar et al., 2015; Zhu et al., 2016) and covert spatial attention (Bowen et al., 2023; Grubb et al., 2013; Kewan-Khalayly et al., 2022; Kewan-Khalayly & Yashar, 2022; Yeshurun & Rashal, 2010). Moreover, the pattern of interference of crowding varies across feature dimensions, such as orientation, color, motion, and spatial frequency (SF) (Greenwood & Parsons, 2020; Yashar et al., 2019). For instance, crowding in orientation often results in the misidentification (misreport) of a flanker as the target, whereas SF crowding leads to an averaging (pooling) of the target with its flankers (Yashar et al., 2019).

Despite these variations across features, a consistent characteristic of crowding is its increase with eccentricity (Pelli & Tillman, 2008; Strasburger, 2020). Crowding can occur within the fovea (Lev et al., 2014; Siman-Tov et al., 2019), and even the foveola (Clark et al., 2020) – the central 1º of the retinal region where visual acuity peaks – but its effects are more pronounced outside the fovea. The critical spacing required to avoid crowding scales with eccentricity (Bouma, 1970; Kurzawski et al., 2023). Yet, the relation between crowding and the visual periphery is complex. A counterintuitive aspect of crowding is the inner-outer asymmetry (also known as the inward-outward anisotropy); that is, in radial crowding (e.g., when target and flankers are positioned on the horizontal meridian), the outer – more peripheral – flanker generates stronger interference than the inner one - closer to the fovea (Banks et al., 1977; Bouma, 1970; Mackworth, 1965,; Petrov et al., 2007; Petrov & Meleshkevich, 2011; Shechter & Yashar, 2021). However, whether and how the inner-outer asymmetry occurs with various objects and feature dimensions is still unknown. For example, in letter crowding, observers report the inner letter instead of the target – a finding interpreted as a reversal of the typical inner-outer asymmetry (Strasburger, 2020; Strasburger & Malania, 2013).

Various theories have been proposed to explain the inner-outer asymmetry, including the scaling of cortical distance (Motter & Simoni, 2007; Pelli, 2008) and receptive field size (Dayan & Solomon, 2010) with eccentricity, and attentional biases toward the visual periphery (Petrov & Meleshkevich, 2011). A recent study employing a mixture modeling approach with an orientation estimation task has shed light on this issue. Shechter and Yashar (2021) used a three-Gabor radial display and asked observers to estimate the target orientation (by adjusting a probe in a continuous orientation space). In one case, the target was the Gabor at the middle of the triad, and in another case, the target was the outmost Gabor. When the target was in the middle of the triad, observers misreported the outer flanker as the target. Interestingly, when the target was defined as the outmost item, the interference – i.e., misreporting the central item (now a flanker) as the target – was substantially reduced (Shechter & Yashar, 2021). Given the constant cortical distance between the middle and outer items in these conditions, these findings challenge explanations based solely on cortical distance. Spatial attention also alters the inner-outer asymmetry with Gabor orientation and T-shape tilt stimuli; cueing attention towards the inner flanker reduces the asymmetry, whereas cueing the outer flanker exacerbates it (Kewan-Khalayly & Yashar, 2022).

Together, these two studies (Kewan-Khalayly & Yashar, 2022; Shechter & Yashar, 2021) suggest that in crowded displays, observers may assign greater sampling weights to the visual periphery, affecting misreport rates. However, crowding can also lead to averaging errors (Yashar et al., 2019), i.e., pooling target and flanker features together (Freeman et al., 2012; Harrison & Bex, 2015; Parkes et al., 2001). However, it is unknown whether, beyond misreport errors, this bias towards the periphery also influences the pooling of feature information.

In the current study, we explored this question by examining the independent contribution of each flanker in various crowded objects and features, such as Gabor orientation and SF as well as T-shape tilt and color. To this end, we reanalyzed continuous estimation report data in various feature domains, originally published by Yashar et al. (2019), focusing on a previously unanalyzed factor. The original study tested the type of crowding errors (pooling vs. substitution) across and between feature dimensions, but it did not consider the relative position of each flanker (inner vs. outer). Here, through continuous report methods and a modeling approach, we evaluated models that apply equal versus variable sampling weights across flankers. Specifically, we compared misreport models with equal versus variable weights and a weighted average model that explains crowding as a pooling of target and flanker information. Based on previous studies, we expect to find a higher misreport rate for the outer flanker than the inner one in Gabor orientation and T-shape tilt and perhaps also in color (Kewan-Khalayly & Yashar, 2022; Shechter & Yashar, 2021). Because observers average the SF values of the flankers and target, we hypothesize that if the more peripheral SF receives higher sampling weights, then the weighted average model will outperform the other models in SF.

## Method

### Behavior

**Fig. 1:**
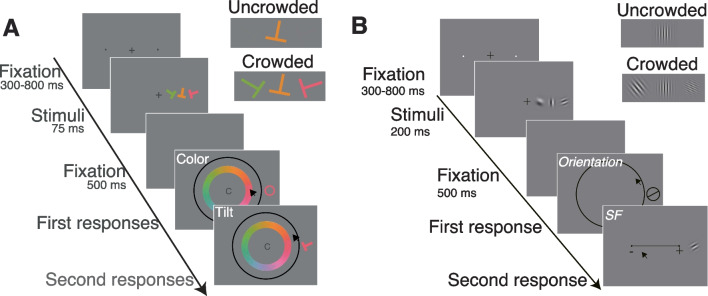
An illustration of the sequence of events within a trial for (**A**) Experiment 1, the target and flankers were colored T-shapes, and observers estimated the tilt and color of the target, and (**B**) Experiment 2, the target and flankers were Gabor patches, and observers estimated the target's orientation and spatial frequency (SF). In each experiment, response order was counterbalanced across trials

We used data from Experiment 1 and Experiment 2 in Yashar et al. (2019). Figure 1A and B depict the sequence of events within a trial for Experiment 1 and Experiment 2, respectively. For each experiment, we collected data from 14 different subjects. We estimated that a sample size of 12 observers was required to detect a crowding effect with 95% power, given a .05 significance criterion on the basis of an a priori power analysis using effect sizes from previous studies (Ester et al., 2014, 2015). We collected data from two more observers in anticipation of possible dropouts or equipment failure. The University Committee on Activities Involving Human Subjects at New York University approved the experimental procedures (IRB-FY2017-182).

Stimuli were presented on a gamma-corrected 21-in. CRT monitor (Sony GDM- 5402; 1,280 × 960 resolution and 85-Hz refresh rate) connected to an iMac. A chin rest was used at a viewing distance of 57 cm. Colors and luminance were calibrated using a SpectraScan Spectroradiometer PR-670 (Photo Research, Syracuse, NY, USA) spectrometer. Eye movements were monitored and recorded by an EyeLink 1000 (SR Research, Kanata, Ontario, Canada) infrared eye tracker. Observers used the mouse to generate responses.

In both experiments, each trial began with a fixation point. After the observer fixated for a random duration between 300 and 800 ms, the target display was presented on the screen for 75 ms. The target was located on the horizontal meridian, either to the left or right of the fixation point, at an eccentricity of 7º visual angle. The target could appear alone (uncrowded) or with two flankers on the horizontal meridian, one on each side (radial crowding), with a center-to-center distance of 2.1º. An eye tracker monitored observer eye fixation during stimulus presentation. Trials in which a fixation break occurred were aborted and rerun at the end of the experiment.

In Experiment 1, the target and flankers were colored T-shaped letter-like stimuli. Tilt and color values ranged from 1º to 360º in a circular space. Colors were chosen from a DKL space and were equiluminant with the background. The target's tilt and color were randomly selected.

Observers (n = 14) estimated the target's tilt and color by positioning the mouse cursor on orientation and color wheels (report order was counterbalanced within observer). The response displays in both experiments remained on-screen until the observer completed both responses. In crowding display trials, flankers differed from the target by either ± 60 or ± 90 in both feature dimensions, resulting in four flanker combinations for each dimension: ± 60/± 60, ± 90/± 90, ± 60/± 90 and ± 90/± 60. Flanker distances within both dimensions were randomly assigned to each flanker, with the restriction that each flanker would have a different direction (±) relative to the target.

In Experiment 2, the target and flankers were Gabor stimuli (a sinusoidal grating enclosed within a 0.325º SD Gaussian envelope and at 85% contrast). The target's orientation (90 values ranging from 1º to 180º) and SF (90 values ranging from one to five cycles per degree (cpd)) were each randomly selected on every trial. SF step size varied depending on the SF values and was determined in a separate test. Namely, to account for the fact that SF discriminability varies as a function SF values (Caelli et al., 1983), we adjusted the scale of the 180 SF steps. This adjustment was based on the variation observed in an SF estimation task in an uncrowded Gabor display in a separate test. We fitted an exponential curve to describe how the variance in SF estimation changed as a function of SF values (see Phase 1 of Experiment 2 by Yashar et al., 2019, for more details). Observers (n = 14) reported the target's orientation and SF by positioning the mouse cursor on an orientation wheel and an SF gradient display. In crowding display trials, flankers differed from the target by either ± 40 or ± 70 in both feature spaces, leading to four flanker combinations in each space: ± 40/± 40, ± 70/± 70, ± 40/± 70 and ± 70/± 40. Flanker distances within both spaces were randomly assigned to each flanker, ensuring each had a different direction (±) relative to the target. For a more detailed description of the *Participants, Apparatus, Procedure, and Design*, see Yashar et al. (2019).

Note that in both experiments, flanker values were randomly assigned to the inner and outer positions, allowing each flanker type (inner or outer) to receive either a negative or a positive value relative to the target.

Here, rather than testing for misreport errors and correlations across dimensions, we investigated the inner-outer asymmetry independently in different feature spaces. To this end, we fitted probabilistic models that quantitatively assess the independent contribution of each flanker in each dimension to individual data.

### Analysis and models

#### Data analysis and preprocessing

We calculated the estimation error in each trial by subtracting the target’s true value from the estimated value (*error* = *report*−*target*). Here we focus on the relative spatial position of each flanker (inner vs. outer) during raw data analysis and model fitting; a factor not considered by Yashar et al. (2019). Initially, for visualization and descriptive statistics, we realigned the direction of errors (positive vs. negative) based on the flanker type (inner vs. outer). This realignment ensured that errors toward the outer flanker would all have the same direction relative to the target, and errors towards the inner target would have the opposite direction. This adjustment was made by setting *error* = −1×*error*, and *flanker* = −1×*flanker* when the outer flanker was negative, and the inner was positive, ensuring the outer flanker was always positive and the inner flanker was always negative. Then, for each observer in each condition, we assessed report bias and report precision by calculating the mean and the inverse of the standard deviation (*std*−1) of the error, respectively.

We analyzed the error distributions by fitting individual probabilistic-mixture models developed from the standard and standard-with-misreporting models (Bays et al., 2009; Zhang & Luck, 2008). We also tested a new probabilistic model that explains error distribution as a weighted average across the different display items.

#### Standard mixture model: Uncrowded and crowded displays

The Standard mixture model (Eq. 1) uses a von Mises (circular normal) distribution to describe the probability density of estimation errors around the target and a uniform component to account for guessing. In this model, the probability of reporting a feature value $P\left(\widehat{\theta }\right)$ is given by:

1 $$P\left(\widehat{\theta }\right)=\left(1-\gamma \right){\phi }_{\sigma }\left(\widehat{\theta }-\theta \right)+\gamma \left(\frac{1}{n}\right)$$

where $\widehat{\theta }$ is the reported feature value, $\theta$ is the actual target feature value, $\gamma$ is the proportion of trials in which observers are randomly guessing (guessing rate), with *n* = 360 for Experiment 1, and *n* = 180 for Experiment 2. ${\phi }_{\sigma }$ is the von Mises distribution with a mean of 0 and a standard deviation *σ* as a free parameter. The model has two free parameters $(\gamma ,\sigma )$.

#### Standard-mixture-with-misreporting models: Crowded displays

The One-misreport model (Eq. 2) introduces a misreporting component to the standard model. This component describes the probability of reporting a flanker as the target. The probability of reporting a feature value $P\left(\widehat{\theta }\right)$ is given by:

2 $$P\left(\widehat{\theta }\right)=\left(1-\gamma -\beta \right){\phi }_{\sigma }\left(\widehat{\theta }-\theta \right)+\gamma \left(\frac{1}{n}\right)+\beta \sum_{i=1}^{m}{\phi }_{\sigma }\left(\widehat{\theta }-{\varphi }_{i}\right)$$

where $\beta$ is the probability of misreporting a flanker as the target, and φ is the actual value of the $i$ *th* flanker, with *m* being the total number of flankers. The variability of the distribution around each stimulus was assumed to be the same. The model has three free parameters $(\gamma ,\sigma ,\beta )$.

In addition to the Standard mixture model and the One-misreport model used in the original study (Yashar et al., 2019), here, we tested models that consider variability between the inner and outer flankers.

The Two-misreport model (Eq. 3) incorporates two misreporting components into the standard model. Each component represents the probability of reporting one of the flankers as the target, such that the probability of reporting a feature value $P\left(\widehat{\theta }\right)$ is given by:

3 $$P\left(\widehat{\theta }\right)=\left(1-\gamma -{\beta }^{In}-{\beta }^{Out}\right){\phi }_{\sigma }\left(\widehat{\theta }-\theta \right)+\gamma \left(\frac{1}{n}\right)+{\beta }^{In}{\phi }_{\sigma }\left(\widehat{\theta }-{\varphi }^{In}\right){+\beta }^{Out}{\phi }_{\sigma }\left(\widehat{\theta }-{\varphi }^{Out}\right)$$

where ${\beta }^{In}$ and ${\beta }^{Out}$ are the probabilities of misreporting the inner and outer flankers as the target, respectively, and ${\varphi }^{In}$ and ${\varphi }^{Out}$ are the actual values of the inner and outer flankers. The model has four free parameters $(\gamma ,\sigma ,{\beta }^{In},{\beta }^{Out})$.

For the SF report, which is not circular, we fitted models using a normal distribution instead of von Mises. Additionally, for SF, the standard model, one-misreport model, and two-misreport model included a bias component, where the distribution around the target ${\phi }_{\sigma ,\mu }$ had a mean *μ* as a free parameter instead of being fixed at 0.

#### Weighted averaged probabilistic model: Crowded display

Furthermore, for all features, we fitted a novel Weighted average model. In this model, the probability of reporting a feature value is:

4 $$p\left(\widehat{\theta }\right)={\phi }_{\sigma ,\overline{\mu }}$$

where ${\phi }_{\sigma ,\overline{\mu }}$ is a Gaussian distribution (von Mises for tilt, color, and orientation, and normal for SF) with a standard deviation $\sigma$ and a mean $\overline{\mu }$, defined as a weighted average:

5 $$\overline{\mu } =\left(1-{\beta }^{In}-{\beta }^{Out}\right)\left(\widehat{\theta }-\theta \right)+{\beta }^{In}\left(\widehat{\theta }-{\varphi }^{In}\right){+\beta }^{Out}\left(\widehat{\theta }-{\varphi }^{Out}\right)$$

where ${\beta }^{In}$ is the weight over the inner flanker, and ${\beta }^{Out}$ is the weight over the outer flanker. The model has three free parameters $(\sigma ,{\beta }^{In},{\beta }^{Out})$.

#### Model-fitting procedure

We used the MemToolbox (Suchow et al., 2013) for model fitting and comparison. To compare models, we calculated the Akaike information criterion with correction (AICc) for the individual fits. We defined the target reporting rate as $Pt=\left(1-\gamma \right)$, $Pt=\left(1-\gamma -\beta \right)$, and $Pt= \left(1-\gamma -{\beta }^{In}-{\beta }^{Out}\right)$, in the standard model, one-misreport model, and two-misreport model, respectively.

#### Multiple comparisons

To correct for multiple comparisons, we applied the Bonferroni correction separately for each tested parameter, i.e., we made corrections based on the number of tests conducted for each parameter type. Given that the number of tests for each parameter type was four, the adjusted significance level was set to *p* < 0.0125.

## Results

### Raw data

**Fig. 2:**
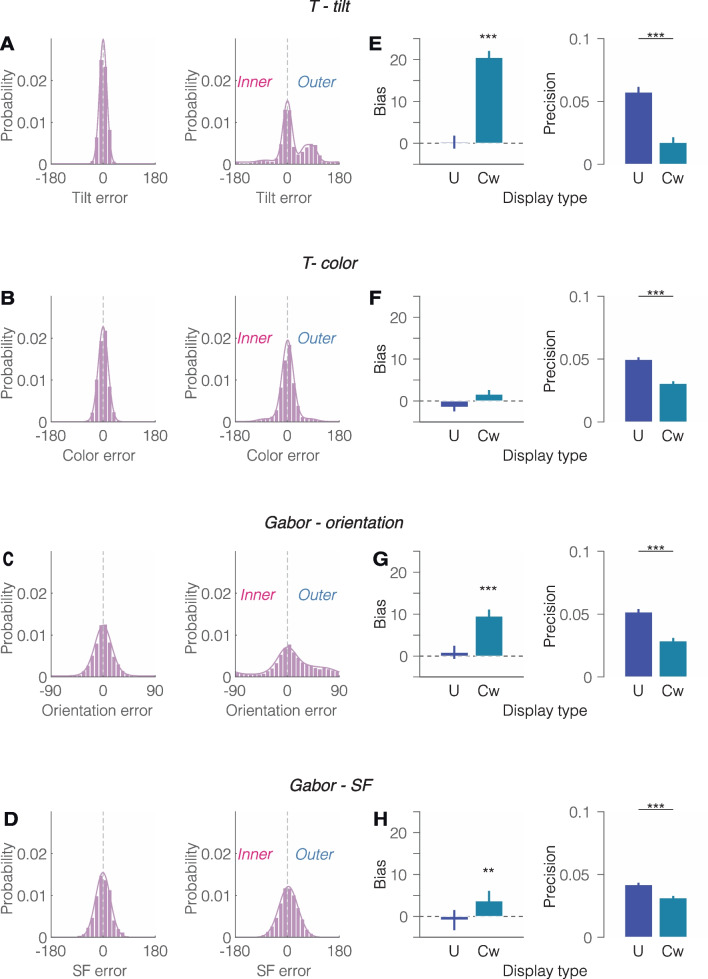
Raw data from Experiments 1 and 2. **A**-**D** show the error distribution for uncrowded and crowded displays in each feature space: **(A)** T-tilt, **(B)** T-color**, (C)** Gabor orientation, and **(D)** Gabor SF**.** For visualization in crowded display trials, error direction was aligned such that all positive values are towards the outer flanker and all negative values towards the inner flanker. Solid lines represent fitted models: two-misreport with bias for tilt and color, two-misreport for orientation, and weighted-average for SF. **E**-**H** depict mean bias (average error) and precision (*sd*^−1^) for (**E**) T-tilt, (**F**) T-color, (**G**) Gabor-orientation, and (**H**) Gabor-SF. U = Uncrowded, and Cw = Crowded. Error bars represent ± within-subject SEM. ** *p* < 0.01, *** *p* < 0.001

Figures 2A-D illustrate the error distribution for each display condition (uncrowded vs. crowded) across tilt and color in Experiment 1, and orientation and SF in Experiment 2. In crowded trials, the distribution skewed towards the outer flanker (positive values). To evaluate the effect of crowding on the raw error distribution, we analyzed bias and precision for each feature space (Fig. 2E-H). In uncrowded display, bias was not significantly different from zero in T-tilt, T-color, Gabor-orientation, and Gabor-SF, *t*(13) = 0.61, *p* = 0.55, Cohen’s *d* = 0.16; *t*(13) = -1.89, *p* = 0.082, Cohen’s *d* = -0.50; *t*(13)=1.21, *p* = 0.246, Cohen’s *d* = 0.32; *t*(13) = -0.32, *p* = 0.752, Cohen’s *d* = -0.09, respectively.

In crowded display trials, bias was significantly larger than zero (indicating a skew towards the outer flanker) for T-tilt, Gabor-orientation, and Gabor-SF, *t*(13) = 9.71, *p* < 0.001, Cohen’s *d* = 2.59; *t*(13) = 4.55, *p* = 0.001, Cohen’s *d* =1.22; *t*(13) = 3.20, *p* = 0.007, Cohen’s *d* = 0.85, respectively, but not for T-color, *t*(13) = 1.57, *p* = 0.141, Cohen's *d* = 0.42. Precision was significantly higher in uncrowded than crowded display trials for tilt, color, orientation, and SF, *t*(13) = 7.05, *p* < 0.001, Cohen’s *d* = 1.88; *t*(13) = 7.92, *p* < 0.001, Cohen’s *d* = 2.12; *t*(13) = 7.06, *p* < 0.001, Cohen’s *d* = 1.89; *t*(13) = 4.67, *p* < 0.001, Cohen’s *d* = 1.25, respectively.

### Model fitting

#### Model comparisons

**Fig. 3:**
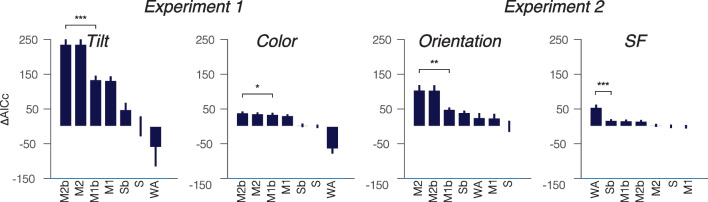
Model comparisons for Experiments 1 and 2. Δ*AICc* for each model across feature spaces, arranged in descending order of fit. Models are denoted as S = Standard Mixture, M1 = One-Misreport, M2 = Two-Misreport, Sb= Standard with Bias, M1b = One-Misreport with Bias, M2 = Two-Misreport with Bias, WA = Weighted Average. Error bars represent ±1 within-subjects SEM. * *p* < 0.05, ** *p* < 0.01, *** *p* < 0.001

For each feature space, to compare models, we calculated Δ*AICc* by subtracting the AICc of each model from the *AICc* of the Standard mixture model, which served as the baseline. The higher the Δ*AICc* (lower *AICc*), the better the model performed. Figure 3 displays the mean Δ*AICc* for individual fits of each model across feature spaces. To test our main hypothesis, we performed a paired t-test between the best-fitted model with variable weights (e.g., Two-misreport, Two-misreport with bias, or Weighted average) and the best-fitted model with equal or no weights (e.g., One-misreport, One-misreport with bias, and Standard mixture).

In Experiment 1, for T-tilt, the two-misreport models outperformed all others, and specifically, the Two-misreport with bias model was significantly better than the One-misreport with-bias model, *t*(13) = 5.49, *p* < 0.001, Cohen’s* d* = 1.47, indicating that orientation misreport rates varied across the inner and the outer flankers. For T-color the Two-misreport with bias model outperformed all other models and was significantly better than the One-misreport with bias model, *t*(13)=2.38, *p* = 0.033, Cohen’s *d* = 0.64.

In Experiment 2, for Gabor-orientation, two-misreport models outperformed all others, and the Two-misreport model was significantly better than the One-misreport model, *t*(13) = 3.64, *p* =0.003, Cohen’s *d* = 0.97. Notably, for Gabor-SF, the Weighted Average model outperformed all others, and was significantly better than the Standard-mixture model, *t*(13) = 5.93, *p* < 0.001, Cohen’s *d* =1.59.

Thus, for T-tilt and Gabor-orientation models with two-misreport components indicate variability in misreport rates between the inner and outer flankers. In contrast, for Gabor-SF the Weighted average model suggests a variable weighting pooling process, potentially reflecting differences in averaging weights across presented items. However, further analysis of fitted parameters is necessary to ascertain whether the variability in misreport rates and weighted averages indeed reflects inner-outer asymmetry.

### Analysis of model parameters

#### Bias and SD

**Fig. 4:**
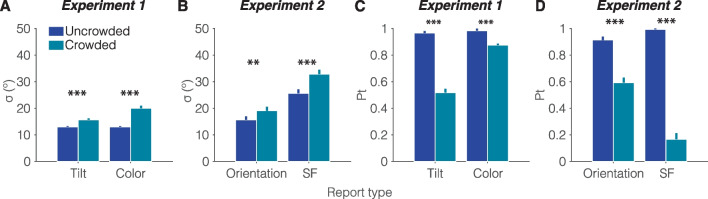
Impact of crowding on model parameters in Experiments 1 and 2*.* **A-B** show mean fitted Gaussian-distribution SD (σ) and **C-D** depict target report rate (*Pt)* as functions of display type: U = uncrowded and Cw = crowded. Error bars represent ±1 within-subjects SEM. ** *p* < 0.01, *** *p* < 0.001

Analyzing the best-fitted model parameters in each space revealed significant differences. The bias component was significantly different from zero for T-color, *M* = 1.27, *SE* = 0.41, *t*(13) = 3.09, *p* < 0.01, Cohen’s *d* = 0.83, but not for T-tilt, *M* = 0.48, *SE* = 0.35, *t*(13) = 1.40, *p* = 0.184, Cohen’s *d* = 0.38. Figure 4 illustrates the Gaussian SD ($\sigma$) and target report rate (*Pt*) as functions of display type across feature spaces. Crowded displays increased *σ t*(13) = 5.76, *p* < 0.001, Cohen’s *d* = 1.54; *t*(13) = 7.34, *p* < 0.00, Cohen’s *d* = 1.96; *t*(13) = 3.62, *p* = 0.003, Cohen’s *d* = 0.97; *t*(13) = 5.04, *p* < 0.001, Cohen’s *d* = 1.35; for T-tilt, T-color, Gabor-orientation, and Gabor-SF, respectively, and reduced *Pt, t*(13) = −11.99, *p* < 0.001, Cohen’s *d* = -.21; *t*(13) = -6.53, *p* < 0.001, Cohen’s *d* = -1.75; *t*(13) = -7.79, *p* = 0.001, Cohen’s *d* = -2.08; *t*(13) = -15.87, *p* < 0.001, Cohen’s *d* = -4.24; for tilt, color, orientation, and SF, respectively. In sum, report bias was significant for T-color; however, the magnitude of the effect was less than 2º. More importantly, in all feature dimensions, the crowded display reduced performance by decreasing report precision and target report rate.

#### Inner-outer weights

**Fig. 5:**
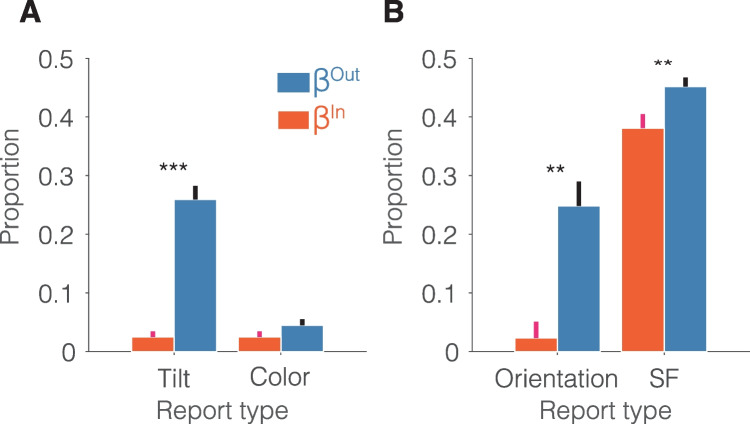
Inner-outer effect as reflected by fitted model parameters in Experiments 1 and 2. Probability of misreporting (**A**: T-Tilt, T-Color, and **B**: Gabor Orientation) as a target or pooling (**B**: Gabor SF) a flanker with the target, as a function of its eccentricity with respect to the target (inner vs. outer) and feature space. Error bars represent ±1 within-subjects SEM. ** *p* < 0.01, *** *p* < 0.001

Further analysis of the inner-outer asymmetry for each feature space is depicted in Fig. 5, which shows the weights over the outer (${\beta }^{Out})$ and inner (${\beta }^{In}$) flankers. ${\beta }^{Out}$ was significantly higher than ${\beta }^{In}$ for T-tilt, Gabor-orientation, and Gabor-SF, *t*(13) = 8.59, *p* < 0.001, Cohen’s *d* = 2.3; *t*(13) = 4.19, *p* = 0.001, Cohen’s *d* = 1.12; *t*(13) = 3.46, *p* = 0.004, Cohen’s *d* = 0.93, respectively, but only marginally so for T-color, *t*(13) = 1.88, *p* = 0.083, Cohen’s* d* = 0.5. Table 1 summarizes these findings across all feature dimensions. These findings indicate that in either misreport and averaging errors observers overweighted the more peripheral outer flanker.

**Table 1:** Comparison of best-fitting models and inner-outer asymmetry across all feature dimensions

Feature	Model	Asymmetry effect size
T-Tilt	Two-misreport	Large
T-Color	Two-misreport	Medium
Gabor-Orientation	Two-misreport	Large
Gabor-SF	Weighted average	Large

## Discussion

This study examines the presence and nature of inner-outer asymmetry within each feature dimension and across various types of crowding errors, such as misreports and averaging. Through modeling the estimation error distributions in four distinct feature dimensions – specifically, T-tilt, T-color, Gabor orientation, and Gabor SF – we quantitatively evaluated the contribution of each flanker to the pattern of errors observed in each feature dimension.

Our results demonstrate that the inner-outer asymmetry – a hallmark characteristic of crowding – can be explained by asymmetric sampling weights with a bias for the more peripheral item, across various feature spaces (T-tilt and color, and Gabor orientation and SF) and crowding patterns (misreports or pooling). However, the magnitude and outcome of these sampling processes vary depending on the feature space. For T-shape tilt and Gabor orientation, observers frequently misreported the tilt and orientation of the outer flanker as the target. For color, observers also misreported the color of the outer flanker more often than the inner flanker, but the misreport rate was overall low, and the asymmetry was not significant. Intriguingly, for SF, observers performed a weighted averaged of all presented SFs, but the outer flanker received higher weights than the inner flanker. That is, averaging was biased towards the more peripheral flanker.

### Not all feature dimensions behave the same way

Yashar et al. (2019) showed that, even with identical objects, the effect of crowding varies depending on the feature dimension. An analysis of precision effects (the inverse of the SD of the error distribution – see Figs. 2E and F) indicates the largest interference with T-tilt and Gabor orientation, followed by T-color, and the smallest for Gabor SF. Moreover, when we arranged the error distribution such that positive values are towards the outer flanker and negative ones towards the inner flanker, there is a consistent bias towards the outer flanker across all feature spaces (Figs. 2A-D). Modeling explains these results as due to the heavier weighting over the outer flanker. Indeed, models with two and differential misreport rates of flankers substantially outperformed models with a single and equal misreport rate of flankers. These findings were pronounced for T-tilt and Gabor orientation and SF, and only slightly so for T-color.

Crowding and the magnitude of the inner-outer asymmetry were more robust for T-tilt and Gabor-orientation than for T-color and Gabor-SF stimuli. These findings are consistent with previous studies that compared crowding across various dimensions (Kewan-Khalayly & Yashar, 2022; van den Berg et al., 2007; Yashar et al., 2019). For instance, van den Berg et al. (2007) reported that crowding effects were significantly greater for orientation and size than for hue and saturation. These results suggest that crowding particularly affects processes related to the representation of orientations and their integration into coherent forms.

The asymmetry effect for color was modest and only marginally significant. One potential explanation is that, consistent with previous studies (Kewan-Khalayly & Yashar, 2022; van den Berg et al., 2007), the overall color-crowding interference, although significant here, is relatively low. This is reflected in the relatively minor effect of crowding on color precision and bias compared to the effects on tilt and orientation. Nevertheless, here with color, models with a misreport component outperformed all other models.

Interestingly, our modeling of the SF results reveals that averaging in SF crowding leads to weighted averaging with a bias towards the most peripheral (outermost) item. Notably, the proportion of averaging weights over the flankers was relatively high. This may be because in many trials, the target feature values were equal to the average of two flanker values (i.e., flankers were -40/+40 or -70/+70), where simple averaging would lead to target reports. This could lead the model to overestimate the proportion of weights over the flankers. However, this can only explain the overestimation of both flankers and cannot account for the bias towards the outer flanker. Yet, modeling detected a higher averaging weight over the outer flanker, consistent with the higher misreport rate of the outer flanker found in T-tilt and Gabor orientation. These findings suggest that the bias towards the outmost item is an inherent characteristic of processing in the visual periphery, regardless of feature dimension.

### Pooling, substitution, and feature binding

A major debate in crowding research is whether its effects are the result of pooling or substitution processes. The former implies a mandatory integration process leading to a new percept comprising both the target and flankers (Harrison & Bex, 2015; Parkes et al., 2001; Rosenholtz et al., 2019). The latter suggests the loss of location information or the entire target information, resulting in the misidentification of a flanker instead of the target (Huckauf & Heller, 2002; Strasburger & Malania, 2013). Population coding models propose that both misreport and averaging errors result from the pooling of target and flanker features, with flanker pooling weights varying as a function of spatial distance (van den Berg et al., 2010; Harrison & Bex, 2015).

Here we demonstrate that both misreport errors and averaging errors can occur, contingent upon the reported feature, within the same display. This is consistent with the findings of Yashar et al. (2019), who demonstrated that these differences are not attributable to performance levels and thus reflect distinct feature-dimension representations and processing. Furthermore, whereas Gabor-orientation errors and Gabor-SF errors correlate, T-tilt errors and T-color errors are uncorrelated, suggesting that crowding occurs after orientation and SF are bound together but before color and tilt (orientation) are bound. Nonetheless, regardless of feature errors and feature-binding, there is a notable sampling bias towards the visual periphery in crowding.

### Possible neurobiological accounts

There is debate over the neurobiological processes and brain areas that underlie crowding; some supporting the receptive field view (Freeman & Simoncelli, 2011; Greenwood et al., 2023) and others the cortical distance view (Kurzawski et al., 2024; Pelli, 2008). The sampling bias towards the more peripheral element, as we show here, has implications for neurobiological accounts of crowding. According to the receptive field size view, variations in crowding across the visual field and the inner-outer asymmetry can be explained by differences in overlapping receptive fields (Dayan & Solomon, 2010; Greenwood et al., 2023). As receptive field size increases with eccentricity, more receptive fields tend to overlap the target with the outer flanker compared to the overlap of the target to the inner flanker. Alternatively, the critical distance view explains the inner-outer asymmetry as due to the smaller cortical distance between the outer flanker and the target compared to the cortical distance between the inner flanker and the target (Pelli, 2008). However, given that receptive field size is inversely related to cortical distance, the presence of inner-outer asymmetry alone does not clearly distinguish between the receptive field size and the cortical distance accounts.

Nonetheless, the present study, together with recent work, provides compelling evidence supporting the receptive field size hypothesis. First, a recent study showed that the magnitude of crowding interference is contingent on the roles of the target and flanker rather than their spacing. Specifically, when elements are arranged radially with consistent spacing, but their roles as target and flanker are alternated, crowding effects vary – with a larger interference when the target is defined as the middle item and a substantially smaller interference when the target is defined as the outermost item (Shechter & Yashar, 2021). Cortical distance cannot explain this finding because it remains the same across report conditions. Furthermore, the cortical distance view predicts stronger interference between the outer flanker and the target, but it cannot provide a parsimonious explanation for the higher rates of misreporting the outer flanker than the inner flanker as the target (Kewan-Khalayly & Yashar, 2022; Shechter & Yashar, 2021), and as shown here.

By contrast, the receptive field view's assertion of the larger number of overlapping receptive fields over the outer flanker predicts both an advantage in performance when reporting the outmost item compared to the middle one (Shechter & Yashar, 2021) and heavier sampling weights for the outer than the inner flanker (Kewan-Khalayly & Yashar, 2022; Shechter & Yashar, 2021). The present study extends support for the receptive field size view across various feature dimensions, stimulus types, and crowding error types by demonstrating heavier weights toward the outer flanker in T-tilt, T-color, and Gabor-orientation misreport rates, as well as Gabor-SF averaging errors.

A limitation of the present study is that we tested crowding at a single eccentricity and only along the horizontal meridian. Cortical surface size area (Himmelberg et al., 2022; Wandell et al., 2007) and receptive field size and overlap (Benson et al., 2018; Dumoulin & Wandell, 2008) increase with eccentricity. Cortical size area also varies as a function of visual field meridian; specifically it is larger at the horizontal than the vertical meridian, and larger at the lower than the upper vertical meridian (Benson et al., 2021; Himmelberg et al., 2022). Likewise, population receptive fields are larger and may overlap more along the vertical than the horizontal meridian (Himmelberg et al., 2023; Silva et al., 2018). Thus, future studies should test variations in flanker weights across different eccentricities, spacing, and meridians.

A possible neurobiological explanation for the dissociable effects among color, spatial frequency (SF), and orientation may rely on differences in visual pathways. The reduced crowding interference in color compared to orientation may be due to the higher reliance on parvocellular (P) cells during color estimation tasks (Shapley & Hawken, 2011), especially given that all colors were isoluminant and varied only in their chromaticity. Parvo cells have smaller receptive fields and therefore may be less susceptible to crowding interference.

In the case of SF, however, the averaging effect of crowding may be due to the nature of the SF estimation/judgment task. Estimation of SF involves assessing the regularities of luminance change over an area. By nature, this task may benefit from sampling over an extended surface and then averaging the rate of change. In a crowded display, before averaging, observers may have sampled over an extended area that included a flanker (mainly the outer one).

## Conclusions

The present study reveals that the dominance of the peripheral flanker over perception occurs in crowding, regardless of feature dimension (i.e., T-tilt, T-color, Gabor-orientation, and Gabor-SF) and integration processes (i.e., misreport or averaging). This phenomenon can be described by heavier pooling weights over the outer flanker. Future population coding models and population receptive field imaging studies of crowding should aim to explain these variations, for instance, by taking into consideration not only receptive field size but also their overlap.

## Data Availability

The data and materials for all experiments are available via the Open Science Framework at at https://osf.io/q9nyg/.

## Code Availability

Analysis code and models are available via the Open Science Framework at https://osf.io/q9nyg/.
